# Mapping functions in health-related quality of life: mapping from the Achilles Tendon Rupture Score to the EQ-5D

**DOI:** 10.1007/s00167-018-4954-y

**Published:** 2018-04-24

**Authors:** Ay-Yen Hua, Olof Westin, Eric Hamrin Senorski, Eleonor Svantesson, Alberto Grassi, Stefano Zaffagnini, Kristian Samuelsson, Mikael Svensson

**Affiliations:** 1 Department of Orthopaedics, Institute of Clinical Sciences at Sahlgrenska Academy, University of Gothenburg, Gothenburg, Sweden; 2 Department of Orthopaedics, Sahlgrenska University Hospital, Mölndal, Sweden; 3 II Orthopaedic Clinic, IRCCS Rizzoli Orthopaedic Institute, Bologna, Italy; 4 Health Metrics, The Sahlgrenska Academy, University of Gothenburg, Gothenburg, Sweden; 5 Department of Health and Rehabilitation, Institute of Neuroscience and Physiology, The Sahlgrenska Academy, University of Gothenburg, Gothenburg, Sweden

**Keywords:** European Quality of Life-5 Dimension, Achilles Tendon Rupture Score, Mapping, Utility value, Quality adjusted life years, Cost-effectiveness analysis

## Abstract

**Purpose:**

Health state utility values are derived from preference-based measurements and are useful in calculating quality-adjusted life years (QALYs), which is a metric commonly used in cost-effectiveness studies. The purpose of this study was to convert the Achilles Tendon Rupture Score (ATRS) to the preference-based European Quality of Life-5 Dimension Questionnaire (EQ-5D) by estimating the relationship between the two scores using mapping.

**Methods:**

Data were collected from a randomised controlled trial, where 100 patients were treated either surgically or non-surgically for Achilles tendon rupture. Forty-three and forty-four patients in surgical group and non-surgical group completed the ATRS and the EQ-5D alongside each other during follow-up at three time points. Different models of the relationship between the ATRS and the EQ-5D were developed and analysed based on direct mapping and cross-validation. The model with the lowest mean absolute error was observed as the one with the best fit.

**Results:**

Among the competing models, mapping based on using a combination of the ATRS items four, five, and six associated with limitation due to pain, during activities of daily living and when walking on uneven ground, produced the best predictor of the EQ-5D score.

**Conclusions:**

The present study provides a mapping algorithm to enable the derivation of utility values directly from the ATRS. This approach makes it feasible for researchers, as well as medical practitioners, to obtain preference-based values in clinical studies or settings where only the ATRS is being administered. The algorithm allows for the calculation of QALYs for use in cost-effectiveness analyses, making it valuable in the study of acute Achilles tendon ruptures.

**Level of evidence:**

II.

## Introduction

Achilles tendon rupture is a common injury and the incidence has been shown to be increasing over the past few decades [14, 16, 20, 23, 24]. While the previous studies have compared whether surgical or non-surgical treatment is the most beneficial in terms of function and outcome, no significant differences have been observed, except for the risk of re-rupture [9, 18, 31, 32]. The debate with regard to the best treatment for Achilles tendon rupture remains ongoing.

A cost-minimisation analysis of the management of acute Achilles tendon rupture has demonstrated that the overall costs of surgical management are higher than those of non-surgical management [29]. However, to the best of our knowledge, surgical versus non-surgical treatments of acute Achilles tendon rupture have not as yet been put in the context of a cost-effectiveness analysis where the differences in cost are compared with the difference in quality-adjusted life years (QALYs), which can show the cost of each QALY gained by a given treatment.

Cost-effectiveness analysis is being used increasingly to inform decision makers with regard to setting priorities in healthcare. Comparing and ranking treatments based on the cost per gained QALY (the lower, the better) can indicate how to maximise patient health benefits given limited healthcare budgets [17]. QALY is a health outcome metric that combines health-related quality of life (HRQoL) and “quantity” of life (life length). One QALY can be viewed as 1 year lived in the best possible health state. The HRQoL used to calculate QALYs is (typically) based on patients’ self-assessed valuations of different health states and often referred to as a preference-based measurement [28]. Different types of preference-based instrument are used to measure the preference-based HRQoL score. These instruments could be condition-specific, but they are commonly generic, i.e., suitable in theory for all kinds of healthcare treatment, and include the EQ-5D, the six-dimensional health state short form [4] and the Health Utilities Index [15], for example. There is no consensus on which preference-based measurement should be used in cost-effectiveness analyses, although the EQ-5D has become increasingly recognised [5, 30].

The Achilles Tendon Total Rupture Score (ATRS) is a primary patient-reported outcome measurement related to symptoms and physical activity after treating total Achilles tendon rupture. The score is reported to have high reliability, validity, and sensitivity [6, 13, 25]. However, it lacks a preference-based score, i.e., how do patients weight the importance of the different items. As a result, it is not possible to use the ATRS directly to calculate QALYs to assess treatments in cost-effectiveness analyses [25]. This problem has been encountered multiple times in clinical studies [5], where a non-preference-based measurement has been the only suitable health measurement available for the condition in question. To solve this problem, a method known as mapping is being used more and more frequently [5, 7, 21, 22]. Mapping investigates the statistical relationship between a non-preference-based measurement and a preference-based measurement, producing an algorithm (“map”) to be used in the calculation of a preference-based HRQoL score. To make this feasible, the method requires a data set of the source measurement (e.g., ATRS) and the target measurement (e.g., EQ-5D) that have been administered alongside each other to the same patients in the relevant clinical trial [5, 30].

If a statistical association between the ATRS and the EQ-5D can be established, i.e., allowing the ATRS to be directly applicable for cost/QALY analyses, it will be valuable in the assessment of treatment for total Achilles tendon rupture. It was hypothesized that a statistical association between the ATRS and the EQ-5D could be established with mapping as an approach. The purpose of this study was to develop an algorithm to convert the ATRS to the EQ-5D by mapping.

## Materials and methods

### Instruments

#### Achilles Tendon Total Rupture Score

The ATRS has been developed to evaluate patient-reported outcome after the treatment of acute total Achilles tendon rupture. The score consists of ten items focusing on one dimension related to symptoms and physical activity. Each item can be graded on a scale of 0 to 10. A summation of the grades gives a total score, ranging from 0 to 100, where a lower score represents greater physical limitation [25].

#### EuroQoL-5 Dimension Questionnaire

The EQ-5D covers five dimensions, including mobility, self-care, everyday activities, pain/discomfort, and anxiety/depression, and each dimension has one item. Each item is rated by the patient according to a three-level (EQ-5D-3L) or five-level (EQ-5D-5L) scale [10] presenting a five-digit number to reflect health state and the level in each dimension. In this study, the EQ-5D-3L was used; there are 3^5^ = 243 different possible health states from which a preference-based single index (utility value) can be derived. The single index is obtained by comparing the five-digit number with the average health state valuation of a population sample generated with the time trade-off (TTO) method or visual analogue scale (VAS) method. The EQ-5D single index ranges from 0 to 1 (although negative values are possible), with 0 regarded as “equal to death” and 1 as “the best imaginable health state” [10, 12].

### The data set

Data were collected from a randomised controlled trial (RCT) conducted to evaluate patient-reported outcomes after stable surgical repair with the early loading of the tendon in patients with an acute Achilles tendon rupture [26]. One hundred patients (86 men, 14 women; age, 18–65 years) were recruited from a centre in Sweden between April 2009 and October 2010, and randomised to either a surgical (*n* = 49) or non-surgical (*n* = 51) treatment group. Patient-reported outcome was assessed for all patients using the self-rated ATRS and EQ-5D during follow-up at 3, 6, and 12 months after treatment. Twelve patients were excluded because of re-rupture or lost to follow-up. From the surgical group, two patients were excluded before first follow-up and another four after follow-up at 3 months. Four patients were excluded before first follow-up and one after follow-up at 6 and 12 months, in the non-surgical group. A final total of 274 paired ATRS and EQ-5D assessments [26] were then collected in the present study to analyse the statistical relationship between the scores.

Ethical approval was obtained from the Regional Ethical Review Board in Gothenburg, Sweden (Diarienr: 032-09).

### Data analysis

The study utilised a direct mapping approach, which is based on a model of the EQ-5D utility scores (“Dolan tariff” [11]) with the ATRS scores as predictors: ${\text{EQ5D}}\;{\text{score}}=f({\text{ATRS}}).$

As there is no theoretical model to guide the model selection for mapping between the instruments, data mining techniques must be applied to detect a potential algorithm that maps ATRS scores onto the utility scores. A standard approach for choosing between different candidate models is cross-validation [2], where the sample is randomly split into two sub-samples. A regression model is estimated on one sub-sample, referred to as the “training sample”, and the results from that regression are used to predict the outcome in the other sub-sample, referred to as the “validation sample”. The accuracy of the model can be assessed by some correlation measurement between the predicted and actual outcomes in the “validation sample” and the model with the lowest prediction error is then regarded as the best fit.

A modified and more efficient version of the cross-validation approach named *K*-fold cross-validation was used. The full sample is split into *K* sub-samples (often 5 or 10) and *K* − 1 sub-samples function as the “training samples”, while one sub-sample functions as the “validation sample”. The model results from the regressions of the training samples are used to predict the outcome in the validation sample and this is repeated *K* times, where each sub-sample functions as the “validation sample” in one of the repeats [8].

**Fig. 1:**
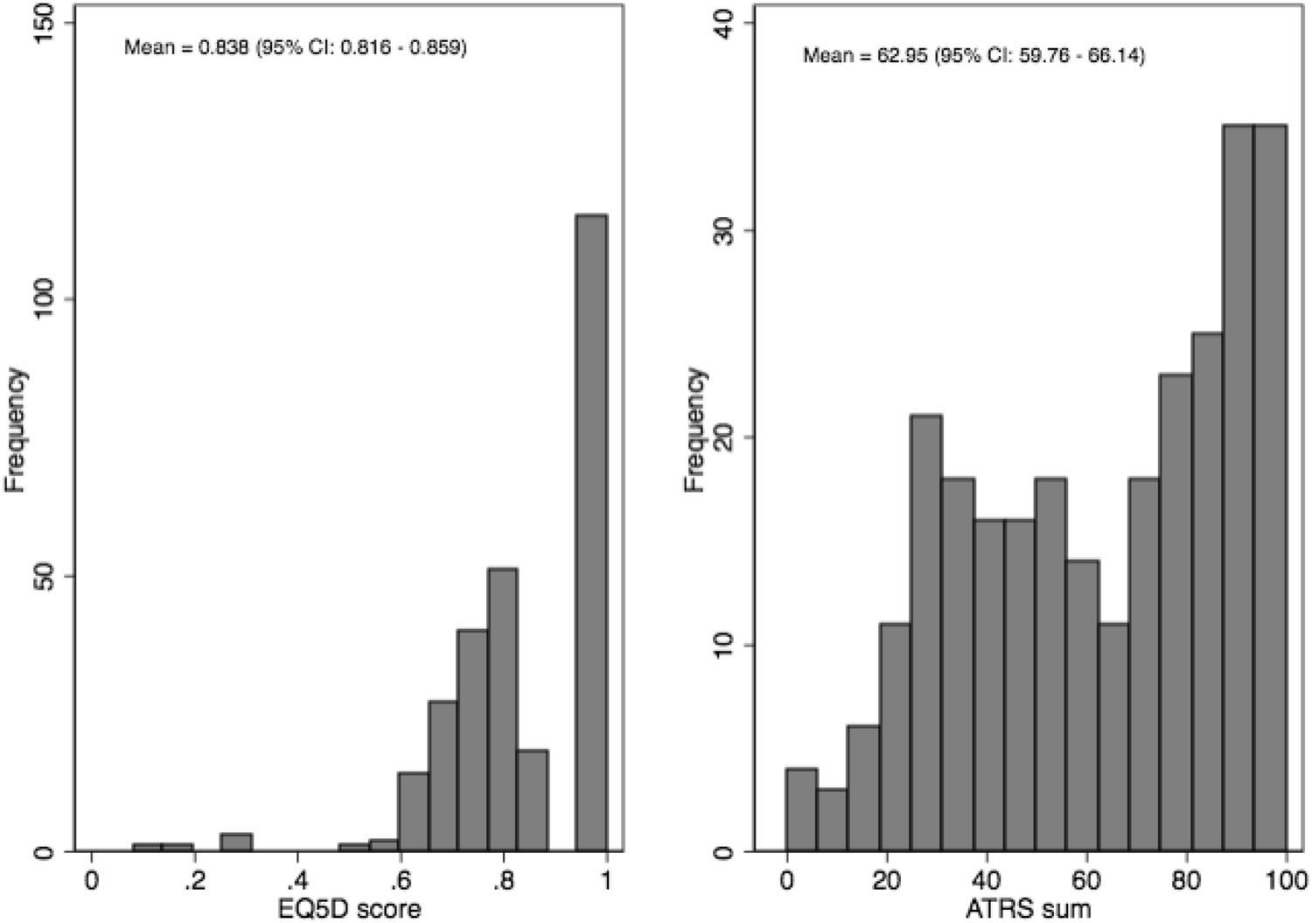
Histogram of EQ-5D and ATRS scores

The model selection is further complicated by the fact that the ATRS data can be summarised in many different ways, e.g., using the summation score of all ten items, as shown in Fig. 1 (from 0 to 100), as the sole predictor, or using the ten items as separate continuous or categorical predictors, or using a subset of the ten items as predictors, etc. Each of these possible alternatives to summarise the ATRS score and function was regarded as the independent variable(s)/predictors (Table 1). For each model specification, we performed the *K*-fold cross-validation and measured the mean of the absolute errors: $${e_i}=|{y_i} - {\overset{\lower0.5em\hbox{$\smash{\scriptscriptstyle\frown}$}}{y} _i}|$$, i.e., the absolute deviation between the observed and predicted outcome in the “validation samples”. The lower the mean of absolute errors (MAE), the better the predictive accuracy of the model.

**Table 1:** Different model specifications

Model name	Variable(s) attempting to predict EQ5D scores	Mean absolute error
Model A	The ATRS sum score (from 0 to 100)	0.0847
Model B	Each of the ten ATRS items as separate continuous predictors (each scored from 0 to 10)	0.0944
Model C	Each of the ten ATRS items as separate predictors but treated as categorical variables	0.1329
Model D*	ATRS items four, five, and six as predictors and treated as categorical variables	0.1509
Model E*	ATRS items four, five, and six as predictors and treated as continuous variables	0.0840

Table 1 shows that model E performed best, with a slightly smaller mean absolute error than the most basic model A. Model E is also a somewhat parsimonious model, including three items from the ATRS to explain EQ-5D scores: items four, five, and six, based on a stepwise regression were the three most statistically significant (and most influential) items from the ATRS in terms of predicting the EQ-5D score. The items describe experienced limitation due to pain in the calf achilles tendon/foot (item four), during acitivies of daily living (item five), and when walking on uneven ground (item six).

## Results

### Mapping results

Table 2 shows the results from model E, which gave the lowest mean absolute error for the potential models tested. Since the data set is a panel of 100 patients measured at three time points, the standard errors for the violation of independence of observations were adjusted.

**Table 2:** Results from model E

Variable	OLS coefficients (std. err.)	Multilevel model coefficients (std. err.)
ATRS item 4	0.0189* (0.0040)	0.0183* (0.0030)
ATRS item 5	0.0181* (0.0045)	0.0158* (0.0044)
ATRS item 6	0.0119* (0.0036)	0.0129* (0.0038)
Constant	0.4784* (0.0400)	0.4936* (0.0213)
Var (constant)^a^	–	0.0016* (0.0009)
*R* ^2^	0.57	–

*p* < 0.001

^a^Variance of constant

The results using a standard ordinary least squares (OLS) adjusted the standard errors by calculating robust standard errors that were clustered at individual level. As an alternative approach, results are also shown from a multilevel (mixed/hierarchical) model where the fact that the data are nested within individuals is directly considered.

The results show that ATRS item 4 has the largest impact on the EQ-5D score, followed by ATRS item 5 and item 6 (true in both model specifications). The mapping equation from the OLS regression is as follows:

$${\text{EQ5D}}\;{\text{score}}=0.4784+0.0189 \times {\text{ATRS}}\;{\text{item}}\;{\text{4}}+0.0181 \times {\text{ATRS}}\;{\text{item}}\;{\text{5}}+0.0119 \times {\text{ATRS}}\;{\text{item}}\;{\text{6}}.$$

The mapping can thus provide EQ-5D scores in the range of 0.4784 up to a maximum of 0.9674, where the latter would be the score for a patient that scores 10 on each of items four, five, and six on the ATRS. To provide a numerical example, consider a patient with the following ATRS responses to items four, five, and six: 8, 7, and 9; this gives:

$${\text{EQ5D}}\;{\text{score}}=0.4784+0.0189 \times 8+0.0181 \times 7+0.0119 \times 9=~0.{\text{8634}}.$$

### Excel application

To accompany the results in this paper, we developed a simple Excel file that can be used directly to map ATRS results to the EQ-5D scores based on the regression results, as shown in Table 2 (available as an online supplement).

## Discussion

The most important finding of the present study was a model for predicting the EQ-5D score from the ATRS. The high *R*^2^ (0.57) indicates a high goodness of fit, even though the model only demonstrated a correlation in three of ten items. In particular, the impact of pain and daily activity was proven to be of specific interest. Brazier et al. [5] reviewed 30 mapping studies for various condition-specific health states, with a total 119 different mapping models. They reported an *R*^2^ of 0.17 for one of the poorer fitting models and an *R*^2^ of 0.51 for the better model when mapping condition-specific measurements onto generic measurements. This suggests a higher level of fitness in this model compared with most other available mapping models.

With its high validity, reliability, and sensitivity, the ATRS is the only patient-reported measurement for the outcome of an acute Achilles tendon rupture [25]. One of the main strengths of mapping the ATRS to a preference-based measurement is that it can be used for cost-effectiveness analysis. There is a little agreement about the most appropriate preference-based measurement for this purpose. Moreover, different preference-based measurements are not guaranteed to generate the same values for the same sample of patients [5]. However, with the extensive use of the EQ-5D and as the most commonly selected target measurement in mapping studies, the EQ-5D has also been the chosen target measurement in the present study.

Although mapping is gaining in popularity, the validity of this method has not been fully addressed. Round and Hawton [27] raised questions about the validity of mapping, arguing that translation from one score to another does not mean that the same health preference is being measured. There are a number of fundamental concerns about mapping, the first of which is the differing sensitivity between the instruments. Generic instruments are designed to measure general health aspects but are insensitive to small health changes. In contrast, condition-specific instruments are inadequate for measuring general health but are sensitive to changes specifically related to the condition of interest. The second is the degree of conceptual overlap between the dimensions measured by the instruments. The less overlap between the dimensions, the weaker the mapping function, and vice versa. Regardless of the degree of overlap, the loss of information associated with dimensions in either of the involved instruments is difficult to avoid when mapping is done. The potential consequences of poor validity are overestimating/underestimating utility values.

Considering these concerns, there is no doubt that mapping to generate utility values is only second best to using preference-based measurements in the first place. However, given that many clinical studies are missing, or were unable to incorporate a preference-based measurement (e.g., not suitable for the relevant condition) and interest in performing QALY-based economic evaluations with clinical studies is growing, mapping is an increasingly used as an alternative solution.

Ideally, the current mapping algorithm may potentially play an essential role in the assessment of Achilles tendon rupture treatment. As already mentioned, the treatment for acute Achilles tendon rupture is either surgical repair or non-surgical treatment. No significant differences in terms of symptoms, function, or result have been shown in the previous studies [9, 18, 31, 32]. There is a reduced risk of re-rupture with a surgical repair (3.1–3.5%) in comparison with non-surgical (12.6–13%) treatment [3, 19], but the downside to this is a higher rate of complications such as infections and adhesions [1]. As the benefits are comparable, there is still no consensus on the best treatment for acute Achilles tendon rupture. However, the current mapping algorithm makes it feasible for researchers and medical practitioners to estimate a utility value for QALY calculation in clinical studies or settings in patients with an acute Achilles tendon rupture where the ATRS is being administered. As a cornerstone of economic analysis, the QALY enables the measurement of economic benefits between healthcare interventions, while incorporating the impact on quality and quantity of life. Given that there are two comparable treatment options for Achilles tendon rupture, using QALYs to measure benefits in cost-effectiveness analyses for both may provide important input in clinical practice, as well as in political decision-making. As a result, the mapping algorithm developed in this study is potentially valuable when assessing the treatment of acute Achilles tendon ruptures.

It should be noted that the mapping algorithm presented in this study will only be applicable for fairly healthy patients with an EQ-5D of 0.47 as the lowest possible score. This is expected, as the analysis is performed on a sample with a high EQ-5D score. It remains to be determined whether the algorithm is applicable to patients with a poorer health state, i.e., by repeating the experiment on a sample with lower EQ-5D scores.

## Conclusions

Utility values are best obtained directly using preference-based measurements, while deriving them with mapping is an alternative solution in clinical trials where only non-preference-based measurements are available. In this study, a mapping algorithm between the ATRS and the EQ-5D was developed, thus providing a way to perform QALY-based cost-effectiveness analyses of acute Achilles tendon rupture treatment.
